# PD40 - A successful desensitisation to cat in a child with anaphylaxis

**DOI:** 10.1186/2045-7022-4-S1-P40

**Published:** 2014-02-28

**Authors:** Pınar Uysal, Carsten Bindslev-Jensen, Nevin Uzuner, Özkan Karaman, Susanne Halken

**Affiliations:** 1 Department of Pediatrics, Division of Allergy and Immunology, Adnan Menderes University Hospital, Aydın, Turkey; 2 Department of Dermatology and Allergy Centre, Odense University Hospital, Odense, Denmark; 3 Department of Pediatrics Division of Allergy and Immunology, Dokuz Eylul University Medical Faculty, Izmir, Turkey; 4 Hans Christian Andersen Children's Hospital, Odense University Hospital, Odense, Denmark

## Introduction

Allergic sensitization to airborne cat allergens is common and mostly related with asthma and allergic rhinitis. Anaphylactic reactions due to exposure to cats have not been reported. Herein, we report the case of 8-year-old boy who had experienced anaphylaxis after contact with cats and successfully desensitized in the first year of immunotherapy.

## Case report

He was referred with a history of suddenly occurring sneezing, urticaria, respiratory distress, wheezing and sometimes hypotension within 30 minutes after contact with cats. He had experienced those symptoms more than five times with a variety of severity in last two years. His past medical history was insignificant for asthma or any of atopic diseases. Allergen specific IgE against cat was 24 kUA/L, and negative against dog and other furred animal dander by ImmunoCAP assay® (Phaida Diagnostics, Uppsala, Sweden). Skin prick test (SPT) was 5 mm for histamine, 11 mm for commercial extract of cat dander and negative for other animal dander and pollens. With regard to unconvincing history, an open challenge test was performed at hospital setting. Anaphylactic reaction with sneezing, rhinorrhea, diffuse urticaria, coughing and mild hypotension developed within 20 minutes after exposure to cat. A subcutaneous immunotherapy protocol was designed with a built-up phase of 6 months. He had no systemic reaction during immunotherapy except large local allergic reactions at injection site at built-up phase. He was evaluated for desensitization at the first year of immunotherapy by a skin prick test and provocation test without any local or systemic allergic reactions.

## Conclusion

For the first time, by presenting this case anaphylactic reactions mediated by exposure to airborne cat allergens is demonstrated to underline the causative role of inhalant allergens in development of anaphylaxis. Moreover, a successful desensitization to cat allergens in a child with anaphylaxis was established in the first year of immunotherapy.

